# Distractor Detection and Suppression Have a Beneficial Effect on Attentional Blink

**DOI:** 10.1371/journal.pone.0044786

**Published:** 2012-09-12

**Authors:** Jing Zhao, Hong Li, Cody Ding, Antao Chen

**Affiliations:** 1 Key Laboratory of Cognition and Personality of Ministry of Education, School of Psychology, Southwest University, Beibei, Chongqing, China; 2 Research Center of Psychological Development and Education, Liaoning Normal University, Dalian, Liaoning, China; 3 College of Education, University of Missouri, St. Louis, Missouri, United States of America; National University of Singapore, Singapore

## Abstract

**Background:**

Attentional blink (AB) is a phenomenon that describes the difficulty individuals have in reporting the second of two masked targets if the second target (T2) arrives 200–500 ms after the first target (T1). Recent studies explain the AB from cognitive resources limitation to distractors interference. For example, the temporary loss of control (TLC) hypothesis suggests that the AB is conduced by distractors disrupting the input filter for target processing. The inhibition models suggest that the T1+1 distractor triggers a suppression mechanism which could be beneficial for T1 processing but would suppress T2 at short T1–T2 lags. These models consider that the AB is caused by the appearance of distractors. However, in the present study, two methods were taken to help individuals to detect the distractors more effectively. An attenuated AB deficit was found when the distractors could be excluded or suppressed in time. We consider that under an appropriate condition the distractors detection and suppression have a beneficial effect on attentional blink.

**Methodology/Principal Findings:**

Two methods were employed to help individuals to detect the distractors more effectively: enlarging the low-level-physical characteristic difference between targets and distractors (Experiment 1) and restricting the sets of distractors (Experiment 2). Attenuated AB deficits were found as using the above manipulations.

**Conclusions/Significance:**

The present study found when the distractors are detected or identified quickly, they could be effectively suppressed, in order to reduce the interference from the targets and result in a smaller AB deficit. We suggest that the suppression mechanism for distractors have a beneficial effect on AB.

## Introduction

Human attention is limited with respect to time, as demonstrated by tasks with the rapid serial visual presentation (RSVP) of stimuli. In these tasks, a rapid stream of visual stimuli is presented in the center of the screen, typically at a rate of 6–20 items per second. Participants have to monitor two targets in the stream (T1 and T2). The first target (T1) is often identified correctly, but the ability to identify the second target (T2) is impaired when it follows T1 within an interval of 200–500 ms [1], [2]. Raymond et al. [2] have termed this impairment to the second of two sequential targets as an attentional blink (AB).

During the past two decades, many theories have been introduced to explain AB. Most [3], [4], [5], [6], [7] share a high degree of convergence in suggesting that T1 depletes the limited cognitive resources and it is this that underlies AB. For example, Chun and Potter’s [7] two-stage model suggests that to prevent the vulnerable conceptual representation of T1 and T2 generated at Stage 1 decaying or being overwritten by subsequent distractors, the targets must be transferred into the capacity-limited Stage 2 to become encoded/consolidated into working memory. However, when the consolidation of T1 has started, T2 will not reach Stage 2 until the consolidation of the first is completed, leading to the AB. As cognitive resources have been utilized to consolidate T1, no more resources are left to consolidate T2 within a short time period. These theories are described as bottleneck theories and can explain most of the AB effect; however, they cannot adequately explain the Lag 1 sparing effect wherein the identification of T2 which follows T1 directly is not impaired [8].

To overcome this shortcoming, several recent studies have shifted their attention from T1 consumption to distractors interference in the AB [9], [10], [11], [12], [13], [14], [15], [16]. Although some bottleneck theories suggest that there is the interference from the distractors to target, they emphasize the limited cognitive resources consumed by T1 as the reason for AB. However, based on distractor-interference perspective, the AB is as a result of the distractors and the Lag 1 sparing effect because no distractor appears between T1 and T2. For example, in the study by Di Lollo et al., the AB was found to be was absent when the three letters-targets continuously presented, but the effect reappeared if a digit was inserted in the target string [10]. They suggest that there is an input filter controlled by a central processor that is suitable for target processing. Once T1 is detected, the central processor shifts from controlling the input filter to consolidating T1. If T1+1 is the second target from the same category as T1, the input filter’s configuration remains unaltered. Thus, T2 can pass through and the Lag 1 sparing effect appears. However, if T1+1 is a distractor from another category (for example the digit), because the input filter is no longer under the control of the central processor and under exogenous control, it will be temporarily disrupted by the distractor. In this case, T2 cannot get though the disrupted input filter, leading to an AB. When the consolidating process on T1 is over, the input filter can be endogenously controlled by the central processor again and reconfigured to allow T2 to pass through. This is referred to as a temporary loss of control (TLC) which emphasizes the distractors interference effect in AB.

The results of Di Lollo et al. [10] reveal a failure in resource-limitation accounts and emphasize the important role of distractors interference in AB. Thus, we wonder whether AB deficit could be attenuated if the distractors could be well suppressed. Martens and colleagues [11], [12], [13], [14] suggest that some individuals such as non-blinkers (approximately 5% of the population) do not show an AB effect in the alphanumeric AB task because they can easily distinguish the digits from the letters based on their well learned alphabetic and numeric category sets, which effectively suppresses these digit distractors. However, most participants (approximately 95% of the population) have no such well learned alphabetic and numeric category sets. Then, how can these participants effectively suppress the digit distractors to reduce the AB deficit? In this study, we consider two ways in which participants may effectively suppress the digit distractors.

In the first experiment, we add the extra source of categorical information (i.e., the presence of a color in the digit distractors). We expect that the extra dimension potentially increases distance between letter and digit categories, and then letter targets can be distinguished from colored digit distractors at an early processing stage, possibly on the basis of perceptual features. Under this condition, fewer cognitive resources may be needed for suppressing the distractors, and the interference with the targets processing is reduced and more cognitive resources could be used for processing T2. Although similarity models [7], [17] would predict an attenuation of the AB in the similar way as in the first experiment, similarity models only consider the role of interference in the AB. However, we not only emphasize the role of distractors interference in AB but also emphasize the more efficient use of cognitive resources. In addition, we expect that TLC hypothesis cannot predict the results from the first experiment because once T1 is followed by a distractor from another category the input filter will be disrupted. In our first experiment, the extra color dimension could potentially increase distance between letter and digit categories, damaging the input filter and making T2 pass harder. Thus, according to the TLC hypothesis, the colored digit manipulation should increase but not decrease the AB deficit.

In the second experiment, we attempt to reduce the candidate digit distractors so that participants may suppress the digit distractors effectively. Landauer and Freedman [18] found the average time required for category recognition was greater for larger categories. Thus, when the digit category set is minimized, participants could more quickly detect the digits as distractors. Although participants could not exclude the distractors on the basis of perceptual features as in Experiment 1, they could suppress the distractors after several processing steps. Under this condition, since the digit distractors could be more quickly and easily suppressed, the cognitive resources could quickly be reused in processing following targets. The TLC hypothesis cannot explain this possible result because this manipulation does not change the attribute of distractors.

The inhibition models, such as the gating theory suggested by Raymond et al., [2] and boost and bounce theory suggested by Olivers and his colleagues [9], [19], also emphasize on distractor suppressing. However, these models contend that post-T1+1 stimuli, including T2 at short T1–T2 lags, are all suppressed in order to protect T1 processing, which leads to AB. Although we also emphasize the suppression mechanism, we expect that the suppressing is only for distractors but not for T2. The suppression mechanism will be beneficial for reducing the AB deficit but not producing it.

## Experiment 1

In Experiment 1, a within-subject design was used. In the control condition, the basic alphanumeric AB paradigm was used in where the letters were the targets and the digits were the distractors. As letters and digits share some line segments and normal participants have no well learned alphabetic and numeric category sets, they cannot distinguish the targets and the distractors easily. In the experimental condition, the distractors were changed from the digits into colored digits and black digits in colored circles, in order to help participants discriminate the distractors more easily. We consider that the colored digits and black digits in colored circles could be better identified as distractors than black digits. Consequently, distractors in the experimental condition could be more efficiently suppressed, resulting in a smaller AB deficit.

### Participants

Fifteen university students (7 males, all right-handed and blinkers, aged 20–24 years) participated in the experiment. All reported normal or corrected-to-normal vision.

### Stimuli Procedure and Design

**Figure 1:**
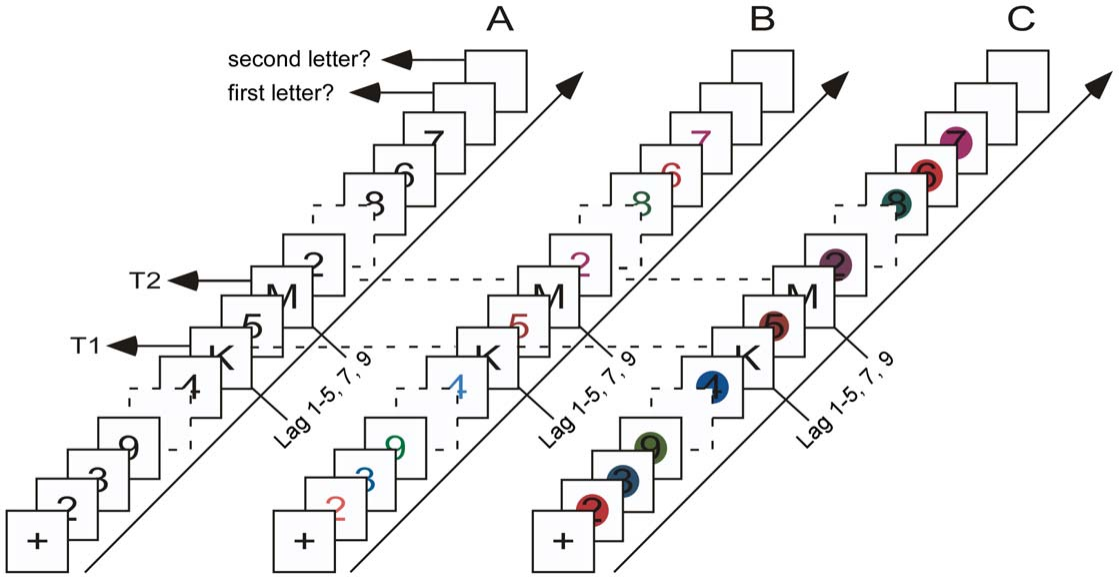
Schematic illustration of the procedures. A) This alphanumeric AB task was used in the control conditions of two experiments, B) this AB task was used in colored digits experimental condition of Experiment 1, C) this AB task was used in black digits in colored circles experimental condition of Experiment 1.

The generation of stimuli and response recording were done with E-Prime 1.1 (SP3). The basic alphanumeric AB paradigm was used. Black letter and digit stimuli were set in 30 point Courier New font and presented on a white background. The stimuli were located in the center of a 17 inch monitor, and viewed at a distance of 60 cm. Each trial started with a fixation presented for 1,000 ms in the center of the display followed by a rapid serial presentation of 11 to 21 digits. Each digit was presented for 50 ms, followed by a blank screen for 30 ms. In each trial, two of the digits were replaced with letters. The second target (T2) was presented with four to six temporal positions from the end of the stream. T2 was the first, second, third, fourth, fifth, seventh or ninth item following T1, corresponding to lags of 80, 160, 240, 320, 400, 560 and 720 ms (i.e., it was presented at lags 1, 2, 3, 4, 5, 7 and 9, respectively). At the end of each trial, participants could report the two targets in any order by pressing the corresponding keys on the keyboard. After 1,000 ms, a new trial started. In both the control condition and the experimental condition, T1 and T2 were randomly chosen from 21 letters without replacement (except for I, O, Q, S and Z). In the control condition (see Figure 1A), the distractors were randomly chosen from eight black digits (except for 1 and 0), whereas in the colored digits experimental condition (see Figure 1B) the distractors were randomly chosen from eight colored digits that were the same size as black digits in the control condition, and in the black digits in colored circles experimental condition (see Figure 1C) the distractors were randomly chosen from eight pictures which were black digits in colored circles and the same size as the letters. The three conditions were presented in separate blocks, each consisting of 189 experimental trials preceded by 14 practice trials. The order of conditions was randomized across participants.

### Results and Discussion

To assess performance on the RSVP task, average T1 and T2 identification accuracy data were submitted to an ANOVA with condition and lag as within-subject factors. Trials on which T1 and T2 were accurately identified but in the wrong order were treated as correct.

**Figure 2:**
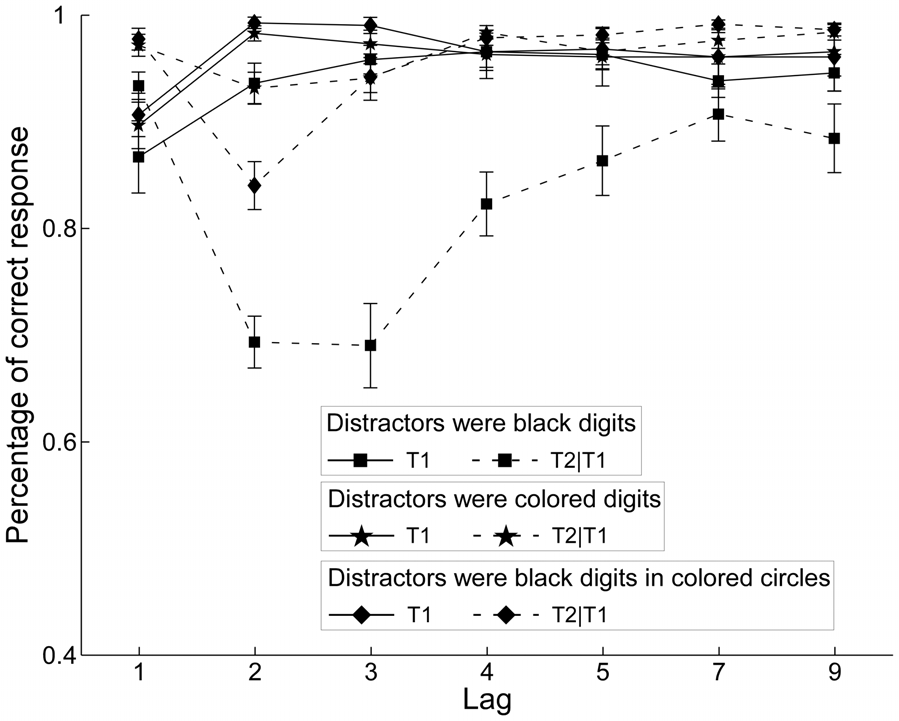
Percentage of correct responses in Experiment 1. Mean percentage correct report of T1 (solid lines) and T2 when T1 was correct (dotted lines) as a function of SOA in the colored digits experimental condition (star symbols), the black digits in colored circles experimental condition (diamond symbols), and the control condition (square symbols) of Experiment 1.

In Figure 2, the solid lines show mean T1 response accuracy in all conditions. Accuracy increased significantly with lag, *F*(6,84) = 25.15, *MSE* = 0.06, *p*<0.001. The effect of the condition was significant, *F*(2,28) = 7.71, *MSE* = 0.07, *p*<0.001. The T1 average correct percentage in the control condition is significantly lower than in the colored digits experimental condition (*p* = 0.013) and in the black digits in colored circles experimental condition (*p*<0.001). The performance between the two experimental conditions was not significantly different, *p* = 0.242. In addition, there was no Condition x Lag interaction (*F* <1).

In Figure 2, the dotted lines show the results for T2 when T1 was identified correctly. There was a main effect of lag, *F*(6,84) = 4.87, *MSE* = 0.08, *p*<0.001. The effect of condition was also significant, *F*(2, 28) = 21.69, *MSE* = 0.11, *p*<0.001. Furthermore, the Lag x Condition interaction was significant, *F*(12, 168) = 13.83, *MSE* = 0.09, *p*<0.001. The drop in accuracy after Lag 1 was shallower in the colored digits experimental condition than in the black digits in colored circles experimental condition and in the control condition. Separate pairwise comparisons revealed that performance in the colored digits experimental condition was better than in the control condition, *p*<0.001. The performance in the black digits in colored circles experimental condition was better than in the control condition too, *p*<0.001. The performance between the two experimental conditions was not significantly different, *p* = 0.118. However, the performance at Lag 2 was better in the colored digits experimental condition than in the black digits in colored circles experimental condition. The performances of two experimental conditions at other Lags did not differ significantly (*F*s <1). And the performances in the control condition at all Lags were significantly worse than in the two experimental conditions.

The AB effect was significantly reduced when the distractors were changed from the black digits into colored digits or black digits in colored circles. Based on these findings, it seemed that the color information from the distractors in the two experimental conditions could be a useful clue to help participants to effectively detect and exclude the distractors in comparison to the black digits in the control condition. One reason may be that the distinguishing process occurs at an early processing stage and the distractors have not been thoroughly processed. Under this circumstance, less cognitive resources are needed to suppress the interference caused by the error processing of distractors.

## Experiment 2

Since Landauer and Freedman [18] found the average time required for category recognition was greater for larger than for smaller categories. We consider that if the digit distractors are from a smaller category, they would be more quickly recognized as distractors than from a larger category. So, in this experiment, we will manipulate on the categorical size to see whether when the digit distractors from a smaller category the AB deficit will be attenuated or not. In both the experimental condition and the control condition, alphanumeric stimuli were used. In the experimental conditions, we reduced the candidate digits and participants were told that the candidate number of digit distractors were reduced from eight (control condition) to five (five candidate digits experimental condition) or three (three candidate digits experimental condition). We consider that the digit distractors in the experimental conditions could be recognized and suppressed more quickly, less interference would be produced and the cognitive resources could be more quickly reused in processing following targets.

### Participants

Fifteen university students (7 males, all right-handed, aged 20–25 years) participated in the experiment. All reported normal or corrected-to-normal vision.

### Stimuli Procedure and Design

The generation of stimuli and response recording were the same as used in Experiment 1. In the control condition (see Figure 1A), participants were told that T1 and T2 were randomly chosen from 21 letters without replacement (except for I, O, Q, S and Z) and the distractors were randomly chosen from eight digits (except for 1 and 0). In five candidate digits experimental condition, participants were told that the targets were randomly chosen from 21 letters without replacement and the distractors were randomly chosen from five digits (2, 4, 5, 7 and 9). In the three candidate digits experimental condition, participants were told that the targets were randomly chosen from 21 letters without replacement and the distractors were randomly chosen from three digits (2, 5 and 9). The three conditions were presented in separate blocks, each consisting of 189 experimental trials preceded by 14 practice trials. The order of the conditions was randomized across participants.

### Results and Discussion

We adopted a significance level of *p*<0.05 and analyzed performance on T1 and T2 separately. The average T1 and T2 identification accuracy data were submitted to an ANOVA with condition and lag as within-subject factors. Trials in which T1 and T2 were accurately identified but in the wrong order were treated as correct.

**Figure 3:**
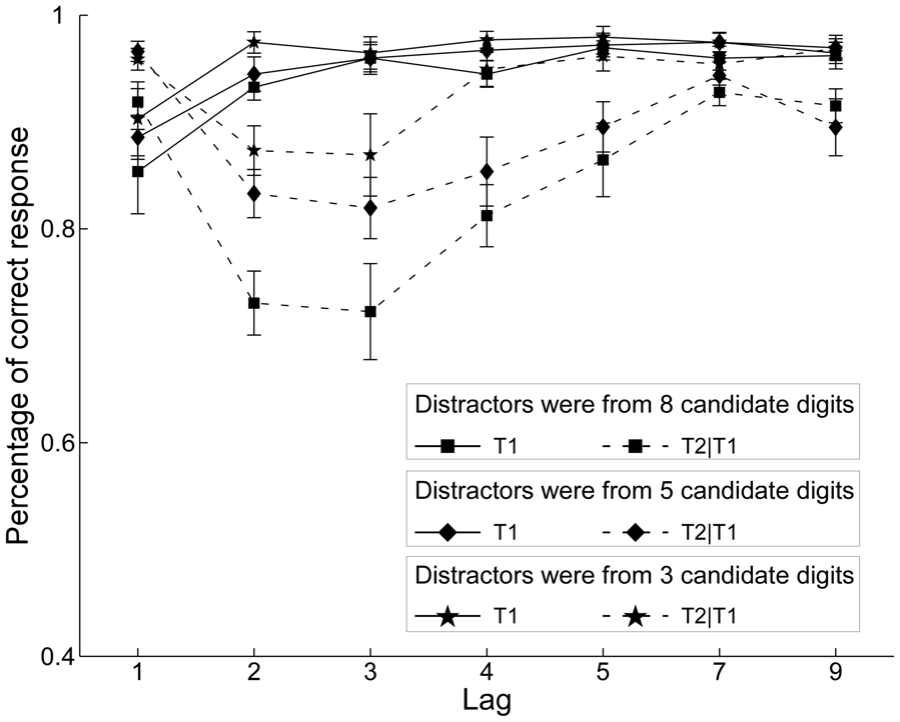
Percentage of correct responses in Experiment 2. Mean percentage correct report of T1 (solid lines) and T2 when T1 was correct (dotted lines) as a function of SOA in the three candidate digits experimental condition (star symbols), the five candidate digits experimental condition (diamond symbols), and the control condition (square symbols) of Experiment 2.

The results for T1 performance (see Figure 3, solid lines) showed that accuracy increased significantly with lag, *F*(6,84) = 28.40, *MSE* = 0.06, *p*<0.001. The effect of the condition was significant, *F*(2,28) = 7.12, *MSE* = 0.05, *p*<0.001. The T1 average correct percentage in the control condition is significantly lower than in the three candidate digits experimental condition (*p*<0.001) and in the five candidate digits experimental condition (*p* = 0.035). Although the performance of the three candidate digits experimental condition looks like better than the five candidate digits experimental condition, the performance between the two experimental conditions was not significantly different, *p* = 0.085. In addition, there was no Condition x Lag interaction (*F* <1).

The results for T2 performance when T1 was identified correctly (see Figure 3, dotted lines) showed a main effect of lag, *F*(6,84) = 47.36, *MSE* = 0.11, *p*<0.001, which demonstrated a classic ‘U’ curve. The effect of condition was also significant, *F*(2, 28) = 59.71, *MSE* = 0.10, *p*<0.001. The Lag x Condition interaction was significant, *F*(12, 168) = 4.68, *MSE* = 0.10, *p*<0.001. Separate pairwise comparisons revealed that performance in the three candidate digits experimental condition was better than in the five candidate digits experimental condition, *p*<0.001. And performance in the five candidate digits experimental condition was better than in the control condition, *p*<0.001. However, the T2 performances at Lag 1 and Lag 2 of the two experimental condition were not significantly different, correspondingly *p* = 0.541 and *p* = 0.111. Performance was firstly promoted heavily at Lag 3 in the three candidate digits experimental condition, which was significantly higher than in five candidate digits experimental condition (*p* = 0.042) and in control condition (*p*<0.001).

In this experiment, only the candidate number of the digit distractors was changed in the three conditions. According to the view of TLC hypothesis, the AB deficit should be the same in three conditions as digits in three conditions had the same chance or ability to disrupt the input filter. However, the result of our experiment showed that the AB deficit gradually minimized when the number of digit distractors reduced from eight to five and three. It seemed that the participants in the restricted set, in comparison to the unrestricted set, could identify the digit distractors more easily and quickly. However, the T2 performance at Lag 2 in two experimental conditions was not significantly different. Comparing with the five candidate digits experimental condition, performance in the three candidate digits experimental condition was promoted heavily at Lag 3. One reason why the there was no a significant difference at Lag 2 may be that distractors in the two experimental conditions of Experiment 2 had no extra different dimension, unlike that in the experimental conditions of Experiment 1. Thus, the distractors could not be detected and excluded at early stage as in Experiment 1. As suggested by Landauer and Freedman [18], the average time required for category recognition was greater for larger categories. Comparing to the five candidate digits experimental condition, the distractor category recognition time was shorter in three candidate digits experimental condition. Participants could more quickly suppress the distractors and the cognitive resources could more quickly be reused in processing the second target. On the other hand, the processing of category recognition needed time even in a very small category. This may result in no significant difference of the T2 performance at Lag 2 in two experimental conditions because the processing of category recognition was not completed when Lag 2 item appeared in both two experimental conditions.

## General Discussion

Throughout the present work, we found that enlarging the low-level-physical characteristic difference between targets and distractors as adding extra source of categorical information - the presence of a color in the digit distractors or using restricted distractor set, the AB deficit was significantly reduced. One common feature between the two experiments was that the two methods were used for reducing the distractors interference with targets processing. However, the specific process of how to reduce the interference was different between the two experiments. In Experiment 1, since the low-level-physical characteristic between the targets and the distractors was enlarged in the experimental conditions, participants could detect and exclude the distractors at early processing stage. Thus, the interference produced by distractors would be reduced and then less cognitive resources were needed in suppressing the interference. In Experiment 2, there was no additional source of categorical information used for detecting and excluding the distractors at early processing stage. Distractors in three/five candidate digits experimental condition and in control condition had the same opportunity to be further processed. These processes may interference with targets processing. However, as less candidate digits existing, the distractors from the smaller category set could be more quickly detected and identified. In this circumstance, cognitive resources could be used to suppress the distractors immediately and reused to process the targets.

Our experimental results suggest that distractors detection and suppression have a beneficial effect on AB. However, the TLC hypothesis [10]cannot well explain our results, as the distractors used in our experiments were all from different categories as the targets, especially the distractors used in the experimental condition of Experiment 1 in which extra source of categorical differences existed. Based on the TLC hypothesis, the AB deficit should be increased or at least keep constant since the distractors were from a more different category. Similarly, the inhibition models [2], [9], [19] cannot well explain our experimental results either, because our results shows that the suppression mechanism induced by distractors only suppress the distractors but not T2.

As been discussed above, we consider that the cognitive resources can be separated into two parts. One part is used to monitor and process the targets, while the other part is used to detect and suppress the distractors. If the targets and the distractors are too similar, it is harder for participants to distinguish the distractors from the targets and the more possibility distractors will get further processing. If so, more interference will be induced and more cognitive resources will be distributed to suppress the interference. Then, fewer resources are left to process the targets. However, if the targets and the distractors are very different from each other (e.g., Experiment 1) or the distractors can be identified and suppressed in a short time (e.g., Experiment 2), more resources can be used to process the targets, resulting in a smaller AB deficit.

Our hypothesis can also explain the Lag 1 sparing effect. As the T1 is followed by T2 directly and there is no distractor between them, in a short time, no cognitive resource needs to be left to suppress the distractor and this can then be used to process T2. However, if there is a distractor between T1 and T2, a part of the resources are busy processing the T1 and other resources are busy suppressing the distractor. Therefore, no more resources are left to process the T2, resulting in an AB deficit. When the T1 processing is over, the resources used for processing targets can be utilized again in processing T2. The percentage of the T2 correct response gradually increases along with the lags, which means that the processing for T1 is gradually over and T2 can obtain more resources.

In conclusion, we suggest that the distractors can be suppressed actively. The AB occurs because the distractors are not well suppressed and interfere with target processing. Although our hypothesis emphasizes the distractor detection and suppression in AB, we also acknowledge the limitation of the cognitive resources. The cognitive resources limitation cannot be surmounted, but can be legitimately distributed to obtain a better cognitive performance.

## References

[1] Broadbent D, Broadbent M (1987) From detection to identification: Response to multiple targets in rapid serial visual presentation. Perception & Psychophysics 42: 105–113. 3627930 10.3758/bf03210498

[2] Raymond JE, Shapiro KL, Arnell KM (1992) Temporary Suppression of Visual Processing in an RSVP Task: An Attentional Blink? Journal of Experimental Psychology: Human Perception and Performance 18: 849–860. 1500880 10.1037//0096-1523.18.3.849

[3] Shih SI (2008) The attention cascade model and attentional blink. Cognitive Psychology 56: 210–236. 17624321 10.1016/j.cogpsych.2007.06.001

[4] Bowman H, Wyble B (2007) The simultaneous type, serial token model of temporal attention and working memory. Psychological Review 114: 38–70. 17227181 10.1037/0033-295X.114.1.38

[5] Fragopanagos N, Kockelkoren S, Taylor JG (2005) A neurodynamic model of the attentional blink. Cognitive Brain Research 24: 568–586. 16099367 10.1016/j.cogbrainres.2005.03.010

[6] Dehaene S, Sergent C, Changeux JP (2003) A neuronal network model linking subjective reports and objective physiological data during conscious perception. Proceedings of the National Academy of Sciences of the United States of America 100: 8520–8525. 12829797 10.1073/pnas.1332574100 PMC166261

[7] Chun MM, Potter MC (1995) A Two-Stage Model for Multiple Target Detection in Rapid Serial Visual Presentation. Journal of Experimental Psychology: Human Perception and Performance 21: 109–127. 7707027 10.1037//0096-1523.21.1.109

[8] Potter MC, Chun MM, Banks BS, Muckenhoupt M (1998) Two Attentional Deficits in Serial Target Search: The Visual Attentional Blink and an Amodal Task-Switch Deficit. Journal of Experimental Psychology: Learning, Memory, and Cognition 24: 979–992. 10.1037//0278-7393.24.4.979 9699304

[9] Olivers CNL, van der Stigchel S, Hulleman J (2007) Spreading the sparing: against a limited-capacity account of the attentional blink. Psychological Research-Psychologische Forschung 71: 126–139. 10.1007/s00426-005-0029-z 16341546

[10] Di Lollo V, Kawahara J, Ghorashi SMS, Enns JT (2005) The attentional blink: Resource depletion or temporary loss of control? Psychological Research-Psychologische Forschung 69: 191–200. 10.1007/s00426-004-0173-x 15597184

[11] Martens S, Korucuoglu O, Smid HGOM, Nieuwenstein MR (2010) Quick Minds Slowed Down: Effects of Rotation and Stimulus Category on the Attentional Blink. Plos One 5: -. 10.1371/journal.pone.0013509 PMC2958832 20975838

[12] Martens S, Dun M, Wyble B, Potter MC (2010) A Quick Mind with Letters Can Be a Slow Mind with Natural Scenes: Individual Differences in Attentional Selection. Plos One 5: -. 10.1371/journal.pone.0013562 PMC2965085 21048954

[13] Martens S, Valchev N (2009) Individual Differences in the Attentional Blink The Important Role of Irrelevant Information. Experimental Psychology 56: 18–26. 19261574 10.1027/1618-3169.56.1.18

[14] Martens S, Munneke J, Smid H, Johnson A (2006) Quick minds don't blink: Electrophysiological correlates of individual differences in attentional selection. Journal of Cognitive Neuroscience 18: 1423–1438. 16989545 10.1162/jocn.2006.18.9.1423

[15] Olivers CNL, Nieuwenhuis S (2006) The beneficial effects of additional task load, positive affect, and instruction on the attentional blink. Journal of Experimental Psychology-Human Perception and Performance 32: 364–379. 16634676 10.1037/0096-1523.32.2.364

[16] Shapiro K, Raymond J (1994) Temporal allocation of visual attention: Inhibition or interference?

[17] Raymond JE, Shapiro KL, Arnell KM (1995) Similarity Determines the Attentional Blink. Journal of Experimental Psychology 21: 653–662. 7790839 10.1037//0096-1523.21.3.653

[18] Landauer TK, Freedman JL (1968) Information retrieval from long-term memory: Category size and recognition time. Journal of Verbal Learning and Verbal Behavior 7: 291–295.

[19] Olivers CNL, Meeter M (2008) A Boost and Bounce Theory of Temporal Attention. Psychological Review 115: 836–863. 18954206 10.1037/a0013395

